# Consensus on Malignant and Benign Tumors in Pediatric Patients with Neurofibromatosis Type 1: On Behalf of the Brazilian Society of Pediatric Oncology (SOBOPE)

**DOI:** 10.3390/curroncol32120664

**Published:** 2025-11-27

**Authors:** Luiz Guilherme Darrigo Junior, Viviane Sonaglio, Sima Esther Ferman, Eliana Caran, Neviçolino Pereira Carvalho Filho, Sidnei Epelman, Vicky Nogueira Pileggi, Julia Lima, Ruth Bartelli Grigolon, Mauro Geller

**Affiliations:** 1 Department of Pediatrics, Ribeirão Preto Medical School, University of São Paulo, São Paulo 14048-900, SP, Brazil; 2 Department of Pediatric Oncology, AC Camargo Cancer Center, São Paulo 01509-010, SP, Brazil; viviane.sonaglio@accamargo.org.br; 3 Department of Pediatric Oncology, Instituto Nacional de Cancer (INCA), Rio de Janeiro 20230-130, RJ, Brazil; sferman@inca.gov.br; 4 Department of Pediatrics, Support Group for Children and Adolescents with Cancer (GRAACC), Federal University of Sao Paulo, São Paulo 04039-001, SP, Brazil; elianacaran@graacc.org.br; 5 Department of Pediatric Oncology, Santa Marcelina Saúde—TUCCA, São Paulo 08270-070, SP, Brazil; oncoped.nevicolino.pereira@tucca.org.br (N.P.C.F.); sidnei.epelman@tucca.org.br (S.E.); 6 Oracle Life Sciences, São Paulo 04710-090, SP, Brazil; vicky.nogueira.pileggi@oracle.com (V.N.P.); julialimanu@gmail.com (J.L.); ruth.bartelli.grigolon@oracle.com (R.B.G.); 7 Department of Immunology and Microbiology, Unifeso, Rio de Janeiro 25964-000, RJ, Brazil; gellerm@ufrj.edu.org

**Keywords:** neurofibromatosis type 1, consensus, pediatric

## Abstract

Neurofibromatosis type 1 (NF1) is an inherited, autosomal dominant syndrome that affects about 1 in every 3000 people worldwide. Early tumor detection is crucial for surveillance and intervention, especially given the potential for serious complications, including visual impairment, skeletal deformities, and malignancy. Therefore, it is essential for pediatricians and other healthcare professionals who provide care to these patients to be aware of all signs, treatments, and management strategies to deliver the best possible care. This study aims to develop a consensus for the diagnosis, treatment, and management of benign and malignant tumors associated with pediatric patients with NF1. Delphi methodology was used to achieve consensus among experts on the diagnostic accuracy, therapeutic efficacy, safety, and surveillance of pediatric patients with NF1. The consensus made 24 recommendations: gliomas in the optic pathway—6 statements, non-optical gliomas—2 statements, plexiform neurofibromas—5 statements, malignant peripheral nerve sheath tumors (MPNST)—6 statements, melanoma—1 statement, juvenile myelomonocytic leukemia (JMML)—1 statement, pheochromocytoma and paraganglioma—2 statements, and gastrointestinal stromal tumors (GIST)—1 statement. This consensus represents the first Brazilian recommendations on malignant and benign tumors in pediatric patients with NF1, providing a framework to standardize and optimize the clinical application for this disease.

## Simple Summary

Neurofibromatosis type 1 (NF1) is a condition affecting children worldwide. Early detection is essential for monitoring and intervention. Currently, there are no specific guidelines for diagnosing, monitoring, and treating tumors in children with NF1. To address this gap in patient care, the Brazilian Society of Pediatric Oncology conducted a systematic review followed by an expert consensus to provide recommendations and evidence on this topic. After two rounds, 24 recommendations were formulated for patient care.

## 1. Introduction

Neurofibromatosis type 1 (NF1) is an inherited, autosomal dominant syndrome that affects about 1 in every 3000 people worldwide [1]. The diagnosis of NF1 is clinical and based on the criteria established by the National Institutes of Health (NIH) Consensus Development Conference in 1987 and revised in 2021 [2].

NF1 is associated with the development of tumors, both benign and malignant, which can appear at an early age. Tumors commonly associated with NF1 include neurofibroma plexiform, optic pathway glioma, malignant peripheral nerve sheath tumor (MPNST), gastrointestinal stromal tumor, breast cancer, leukemia, phaeochromocytoma, duodenal carcinoid tumor, and rhabdomyosarcoma [3].

Identifying tumors is essential for proper monitoring and treatment, mainly because of the risk of serious complications, including vision loss, skeletal deformities, and cancer development [4]. Therefore, it is essential for pediatricians and other specialists who provide care to these patients to be aware of all signs, treatments, and management/monitoring strategies to offer the best care possible. In this context, this study aims to develop a consensus for the diagnosis, treatment, and management of both benign and malignant tumors associated with pediatric patients with NF1.

## 2. Materials and Methods

### 2.1. Study Design

This study used the Delphi methodology to achieve consensus among experts on the diagnostic accuracy, therapeutic efficacy, safety, and monitoring and surveillance of pediatric patients with NF1 to develop consensus-based clinical recommendations for those patients. The Delphi method was chosen for structured expert consensus, which is particularly valuable where high-quality evidence is limited.

### 2.2. Selection of Experts

Experts were selected based on their recognized expertise and experience in the field. The panel invited 65 experts in the first round and 28 in the second round from diverse regions of Brazil and different specialties, including oncology pediatricians, geneticists, neurologists, dermatologists, radiologists, neuroradiologists and plastic surgeons, regardless of their practice setting (public or private). This ensured a wide range of views and experiences in the field.

### 2.3. Questionnaire Development

A working group composed of six medical experts (LGDJ, VS, SEF, EMMC, NPCF, and SS), members of the Brazilian Society of Pediatric Oncology (SOBOPE), and three experts in Delphi methodology (RBG, VNP, and JL) from Oracle Life Sciences, met over three months to review the available literature and develop the questionnaire. To identify the available literature, search strategies were developed in the MEDLINE (via PubMed), LILACS (via BVS), and Cochrane databases in January 2025. Only studies published after 2014 were included in this analysis. Table 1 presents the complete search strategies.

### 2.4. Delphi Procedure

The consensus was structured into the following areas, with the corresponding number of statements:

1. Gliomas in the optic pathway—6 statements
2. Non-optical gliomas—2 statements
3. Plexiform Neurofibromas—5 statements
4. Malignant Peripheral Nerve Sheath Tumors (MPNST)—6 statements
5. Melanoma—1 statement
6. Juvenile myelomonocytic leukemia (JMML)—1 statement
7. Pheochromocytoma and Paraganglioma—2 statements
8. Gastrointestinal Stromal Tumors (GIST)—1 statement

Sixty-five experts received an invitation to the first round of the Delphi process, and 28 in the second round, which was conducted through an anonymous electronic voting system. A list of references supporting each statement was provided.

The Delphi panel was initially planned to be conducted in three steps. However, no additional rounds were necessary since consensus was reached on all statements in the second round. The questionnaire was distributed to the experts via a link, and they indicated their level of agreement with each statement using the 5-point Likert scale. We had a total of three links divided by area of expertise (Table 2). Responses were aggregated, and the distribution of scores was analyzed for each item.

The 5-point Likert scale was used to collect responses:

9. 1 = Strongly Disagree
10. 2 = Disagree
11. 3 = Neutral or Indifferent
12. 4 = Agree
13. 5 = Strongly Agree

### 2.5. Consensus Criteria and Level of Evidence

Consensus was defined as at least 80% agreement among the experts, meaning that 80% of participants would indicate “Agree” or “Strongly Agree” (scores 4 or 5 on the Likert scale) for a given statement. The evidence used in this guideline was classified using the scheme below Table 3 [5]:

## 3. Results

### 3.1. Characterization of Expert Sample

A total of 23 experts participated in the study (Appendix A). Among them, 52.2% were male, and the average age was 49 years (31–69). Most experts worked in both the private and public healthcare systems (56%), while 9% worked exclusively in the private sector, and 35% worked exclusively in the public sector. The average number of patients with NF1 attended by each physician was more than 50 in 56.5% of the sample, between 10 and 50 in 39.2%, with only 4.3% attending fewer than 10 patients yearly.

Regarding the medical specialties, 30.4% were geneticists, 26.2% were pediatric oncologists, 8.7% were dermatologists, 8.7% were surgeons, 8.7% were pediatric neurologists, 8.7% were clinical neurologists, 4.3% were radiologists, and 4.3% were immunogeneticists. Table 4 summarizes all 24 recommendations, including the percentage of consensus and the level of evidence. The recommendations that did not reach consensus in phase 1 can be found in Appendix B.

### 3.2. Optic Pathway Glioma

**Recommendation 1**. It is recommended for all patients newly diagnosed with NF1 should be followed up by an ophthalmologist to evaluate visual acuity, visual fields, pupillary reflexes, eye movements, and optic disc aspect. Ophthalmic follow-up should be annual until early adulthood. (100% agreement on the first round)—Evidence III.

Optic pathway gliomas (OPGs) are a frequent manifestation in children with NF1, affecting 15–30% of pediatric NF1 cases [6]. Glombova et al. analyzed data from 285 children with NF1 followed between 1990 and 2010 and found that 27% had OPGs [7]. Similarly, Prada et al. conducted a 20-year study of 826 NF1 patients aged 1–9 years, identifying OPGs in 18% of cases, with a median age at detection of 3 years [8]. While some OPGs may remain stable or even regress spontaneously, others can lead to significant visual or neurological deterioration, underscoring the importance of early detection and monitoring. The strong association between OPGs and NF1 has sparked debate about the utility of routine vision screening in patients with NF1. However, screening remains challenging due to the variability in clinical presentation and the lack of consensus on optimal screening protocols. A 2015 study by Caen et al. highlighted significant disparities in screening practices among NF1 centers. While most centers perform ophthalmological screening until ages 16–18, others stop at a much younger age. Screening intervals also vary widely, with some centers recommending half-yearly intervals for younger children, as OPGs often progress during early childhood. However, tumor growth can occur after age 6, emphasizing the need for continued monitoring. The recommended ophthalmological examination includes visual acuity (VA) assessment, pupillary reflexes, and fundoscopy [9]. Azizi et al. (2020) identified several risk factors associated with adverse visual outcomes in pediatric NF1-OPG patients, including multiple visual signs and symptoms at diagnosis (adjusted odds ratio [adjOR]: 8.33; 95% CI: 1.9–36.45), abnormal visual behavior (adjOR: 4.15; 95% CI: 1.20–14.34), new onset of visual symptoms (adjOR: 4.04; 95% CI: 1.26–12.95), and optic atrophy (adjOR: 3.73; 95% CI: 1.13–12.53) [4]. These findings highlight the importance of regular ophthalmic screening during infancy and early childhood [4]. Robert-Boire et al., conducted a 15-year review of OPG cases, finding that 42.5% were NF1-related. While 65% of NF1 patients were symptomatic at diagnosis, 15% were asymptomatic, with tumors detected incidentally during routine magnetic resonance imaging (MRI). Interestingly, non-NF1 patients were more likely to present with symptoms (91.3%) than NF1 patients (29.4%). Squinting was more common in sporadic OPGs, while nystagmus was more frequently observed in non-NF1 cases, likely due to larger chiasmatic lesions that can induce hydrocephalus or Parinaud’s syndrome [10]. Kinori et al., in a retrospective observational case series with a follow-up of at least 10 years, provided further insights into the natural history of OPGs in NF1 patients [11]. Most patients (78%) had no visual complaints at diagnosis, but 47% exhibited abnormal optic nerve head appearance (swollen/edematous or pale) on fundoscopic exam, with 25% unilateral and 22% bilateral involvement [11]. Additionally, 31% had other signs attributable to OPGs, such as proptosis (13%) and strabismus (13%), or abnormal visual acuity (31%), defined as moderate to severe impairment (20/40 or worse) [11]. Even asymptomatic NF1 patients often exhibit visual impairment upon detailed examination, underscoring the importance of careful monitoring. Some studies suggest that VA testing has a sensitivity of only 50% in detecting OPGs, with false positives occurring in patients without tumors [12,13]. These limitations are particularly pronounced in younger children, who may struggle with cooperation and focus during testing. Visual field testing (VF), another diagnostic tool, is particularly challenging in children under 3 to 4 years old, and its results should be interpreted with caution for this age group [13,14]. Improved screening methods, including reliable biomarkers and age-appropriate testing protocols, are needed to enhance the early identification of OPGs and optimize outcomes for NF1 patients. In the meantime, regular ophthalmological evaluations and a tailored approach to visual monitoring remain essential for managing this high-risk population.

**Recommendation 2**. It is recommended that pediatric patients with NF1 undergo regular clinical ophthalmologic evaluations, complemented by imaging tests such as optical coherence tomography (OCT), to investigate choroidal and retinal alterations. The evaluation should be performed at least once, according to the clinical and visual evolution of the patient. (100% agreement on the second round)—Evidence III.

Optical coherence tomography (OCT), which measures circumpapillary retinal nerve fiber layer (cpRNFL) thickness, offers an objective alternative to assess damage to the anterior visual pathway. Parrozzan et al. demonstrated that RNFL thickness in pediatric NF1 patients with OPGs correlates directly with VA, establishing a global RNFL thickness cut-off of 76.25 µm to distinguish normal from pathologic VA [15]. This is particularly useful in young children, as approximately 19% struggle with VA testing compliance due to age or attention deficits. Spectra domain optical coherence tomography (SD-OCT) requires minimal cooperation, making it a practical tool for this population. While RNFL thickness can reassure clinicians and families about the absence of significant VA impairment, it should not dictate treatment decisions alone. Instead, it serves to confirm clinical suspicions and guide intervention timing [15].

Jiang et al. highlighted OCT’s utility in young children who cannot reliably perform VA tests, as it objectively measures damage to the anterior visual pathway [16]. Bowman et al. further explored the prognostic value of OCT, finding that mean peripapillary RNFL thickness predicted better final acuity in the better eye (OR 0.799, 95% CI: 0.646–0.987, *p* = 0.038). Conversely, visual symptoms at presentation (OR 0.22, 95% CI: 0.001–0.508, *p* = 0.017) predicted worse final acuity in the more severely affected eye. These findings underscore the value of RNFL thickness in predicting visual outcomes and guiding treatment, especially in children with homonymous hemianopia [17].

Vagge et al. compared the diagnostic accuracy of visual function assessment, and RNFL analysis by OCT in 110 NF1 patients. Global RNFL thickness showed the highest diagnostic accuracy for detecting OPGs (AUC = 0.758). Even in asymptomatic children with normal VA, RNFL thickness remained a reliable indicator of OPGs [13]. OCT’s advantages include widespread availability and minimal cooperation requirements, making it feasible for young NF1 patients. However, its sensitivity remains suboptimal, and MRI remains the gold standard for definitive OPG diagnosis.

**Recommendation 3**. Routine screening with MRI of the central nervous system and optical pathways is recommended for pediatric patients with NF1 and associated symptoms such as recent visual impairment or physical signs such as proptosis, strabismus, nystagmus, persistent headache, precocious puberty, abnormal growth patterns, or other ophthalmological and/or neurological signs and symptoms suggestive of optic tumors. In addition, in regions with limited access to specialized ophthalmologic evaluation, MRI in pediatric patients with asymptomatic NF1 represents an important and safe tool for monitoring these patients. (100% agreement in the second round)—Evidence III.

MRI remains the gold standard for tumor follow-up, but its necessity is increasingly debated. Marsault et al. evaluated the diagnostic performance of unenhanced MRI for monitoring OPGs and found it to have high sensitivity (84–88%) and specificity (91.3–100%) compared to contrast-enhanced MRI. Unenhanced MRI also demonstrated excellent inter-observer agreement (kappa coefficient: 0.87) and good reproducibility for tumor volume measurements. These findings suggest that contrast-enhanced MRI should be reserved for cases where clinical or radiological progression is observed on unenhanced sequences, thereby reducing unnecessary gadolinium exposure while maintaining diagnostic accuracy [18].

Maloney et al. further explored the need for gadolinium-based contrast agents (GBCAs) in the routine surveillance of isolated OPG. Given the frequent use of contrast-enhanced MRI and the excellent overall life expectancy of children with OPGs, these patients are at higher risk of gadolinium tissue deposition and potential long-term complications. The authors hypothesized that GBCA administration is unnecessary for routine MRI surveillance of isolated OPGs, as changes in tumor size, rather than contrast enhancement characteristics, drive clinical management decisions. Their retrospective review supported this hypothesis, showing that routine surveillance MRIs for isolated OPGs do not require gadolinium to guide treatment decisions. [19].

While contrast-enhanced MRI remains a valuable tool for diagnosing and monitoring OPGs, emerging evidence supports using unenhanced MRI for routine surveillance [19]. It is important to note that MRI is costly and often requires sedation or anesthesia in younger children, which limits its use for routine screening [12]. MRI surveillance for asymptomatic children with NF1 is not indicated [20]. This is because the early identification of asymptomatic OPGs has not been shown to significantly alter disease progression or outcomes. For example, Henning et al. reported that among 21 children with OPGs, 33% had no OPG-related symptoms prior to the MRI being performed. Yet, systematic screening did not significantly change management in asymptomatic cases [21]. Trevisson et al. assessed the natural history of OPG in a cohort of unselected patients affected by NF1. The study retrospectively evaluated 414 consecutive patients with NF1, of whom 44.7% underwent brain and orbit MRI [22]. Among these, 34.6% were performed for screening purposes, while 65.4% were conducted due to the presence of neurological, ocular, or other symptoms. OPG was diagnosed in 12.5% of the patients who underwent MRI for screening, compared to 36.4% of those who had MRI due to clinical symptoms (*p* = 0.001). Clinical management was conservative in most cases, with only eight patients requiring therapy, primarily due to visual deterioration. Notably, all patients who underwent screening MRI had normal visual outcomes, further supporting the argument that early detection of asymptomatic OPGs through MRI does not significantly alter prognosis or treatment needs [22]. Prada et al. evaluated the utility of screening brain and orbital MRI scans in a large population of children with NF1 over a 20-year period. Among 826 children screened, 18% were diagnosed with OPGs, with only 2.7% requiring chemotherapy. None of the children identified through MRI alone (without visual symptoms) progressed to vision loss. The detection of non-progressive or spontaneously resolving lesions can lead to unnecessary parental anxiety and increased healthcare costs. The study also highlighted the importance of tumor location, as pre-chiasmatic OPGs were more likely to regress (25%) compared to chiasmatic and post-chiasmatic tumors (10%), which were more frequently associated with visual symptoms and required therapy. The majority of OPGs detected through screening are asymptomatic and do not progress, reinforcing the current recommendation against routine MRI screening in asymptomatic NF1 children [8]. Sellmer et al. further corroborates these findings, reporting that the prevalence of OPG among NF1 patients was highest in children aged 10 or younger and declined with advancing age. The rate of progression over 5 years was 2.4% (95% CI: 0.4% to 16%), while the rate of regression over the same period was 8.9% (95% CI: 2.8% to 26%). These observations suggest that OPGs are common in children but are typically asymptomatic and may sometimes regress spontaneously in late childhood or adolescence. This supports the current practice of limiting frequent follow-up MRIs to patients with symptomatic OPGs or tumors showing growth or mass effect [23]. Similarly, Azizi et al. found that the association between MRI surveillance and a lower risk of visual deterioration was not significant in multivariable analysis (adjOR: 0.77, 95% CI: 0.25–2.34) [4].

While MRI remains indispensable for diagnosing and monitoring symptomatic OPGs, its limitations in cost, practicality, and the lack of evidence supporting improved outcomes in asymptomatic cases have led to a consensus against routine screening in asymptomatic NF1 children. Instead, regular ophthalmological evaluations and targeted use of MRI based on clinical findings are recommended to balance early detection with the avoidance of unnecessary interventions and parental anxiety.

**Recommendation 4**. Biopsy is not recommended for diagnostic confirmation of optic pathway gliomas in pediatric patients with NF1. (100% agreement in the first round)—Evidence III.

The need for a biopsy to confirm the diagnosis of OPGs remains a topic of debate. While a biopsy can provide definitive histologic confirmation, it carries surgical risks and may delay the initiation of treatment. Additionally, it is unclear whether biopsy offers significant clinical benefits, particularly when imaging findings are consistent with low-grade glioma, especially in patients with NF1 [24]. As a result, most centers avoid biopsy in such cases to preserve optic nerve function and rely on MRI findings for diagnosis.

For example, in a study by Doganis et al., the diagnosis of OPGs was confirmed in all children based on MRI findings. However, three atypical patients (two without NF1) had undergone biopsies that revealed histological findings consistent with grade I pilocytic astrocytoma [24]. This highlights that while biopsy may be necessary in atypical or unclear cases, it is not routinely required when imaging strongly suggests low-grade glioma, particularly in the context of NF1.

**Recommendation 5**. An initial approach with vincristine and carboplatin is recommended as the first line of treatment for pediatric patients with NF1 and the presence of symptomatic optic pathway gliomas. (83% agreement in the first round)—Evidence III.

In OPG associated NF1 patients, treatment with carboplatin and vincristine (CV) demonstrated superior efficacy and tolerability compared to non-NF1 patients. Among 127 NF1 patients analyzed, the CV regimen showed a five-year event-free survival (EFS) of 69% ± 4% compared to 39% ± 4% in non-NF1 patients (*p* < 0.001). Overall survival (OS) was also significantly higher in patients with NF1, with a 5-year OS rate of 98% ± 1%, compared to 3% ± 1% in patients without NF1 (*p* = 0.003). This pattern was also consistent for hypothalamic/optic chiasmal tumors, with patients with NF1 having a 68% ± 5% 5-year OS, compared to 38% ± 6% in those without NF1 (*p* < 0.001) [25].

NF1 patients experienced a favorable overall toxicity profile, with less than 5% cumulative grade 3 or 4 toxicity. Notably, NF1 patients exhibited higher tumor response rates, with no deaths or severe adverse events related to chemotherapy during the treatment period [25].

These findings highlight the efficacy and safety of CV chemotherapy in NF1 children, particularly for achieving tumor control and minimizing toxicity. However, the decision to initiate therapy in NF1 patients should be carefully weighed, as the indication for treatment initiation remains a topic of ongoing debate [25].

Ruggiero et al. conducted a study to evaluate the effectiveness of vincristine and carboplatin as a first-line chemotherapy regimen for pediatric low-grade gliomas (LGG), focusing on differences in response between NF1-associated and sporadic gliomas [26]. This retrospective analysis included 60 children, of whom 18 (30%) had NF1. Tumor response was assessed using MRI and categorized into disease reduction, stability, or progression [26]. The results showed that 81.8% of NF1 patients achieved significant disease reduction following chemotherapy, compared to 42.8% of patients with sporadic gliomas, a statistically significant difference (*p* < 0.05). Toxicity was manageable, with no reported treatment-related deaths. NF1-associated gliomas demonstrated a more favorable response to chemotherapy, likely due to their distinct tumor biology, including slower progression and less aggressive clinical behavior compared to sporadic gliomas [26].

**Recommendation 6**. The following therapeutic options are recommended as second-line therapy for pediatric patients with NF1 and the presence of recurrent or refractory optic pathway gliomas: vinblastine, carboplatin, vinorelbine, or bevacizumab, isolated or in combination. (83% agreement in the first round)—Evidence III.

Cappellano et al. reported the efficacy and safety of vinorelbine as a single-agent treatment for progressive OPG in pediatric patients, with a focus on managing unresectable low-grade gliomas (LGGs) while minimizing toxicity. This phase II trial included 23 children, three of whom had neurofibromatosis type 1 (NF1). Vinorelbine was administered intravenously every four weeks for 18 cycles, with response and toxicity assessed at regular intervals [27]. Among the three NF1 patients, one achieved a complete radiological response with normalization of chiasmatic and optic nerve structures, while the other two showed minor response and stable disease, respectively. Toxicities were primarily hematologic, with grade III/IV neutropenia occurring in four patients, and gastrointestinal side effects being mild to moderate [27]. Karla et al. demonstrated the efficacy and safety of bevacizumab-based therapy (BBT), combined with irinotecan, in children with recurrent or refractory LGG. It retrospectively analyzed 16 pediatric patients treated between 2009 and 2013, including 5 with NF1. Eligible patients had experienced disease progression or recurrence following at least one prior line of therapy and were treated with intravenous bevacizumab (10 mg/kg) and irinotecan (125–150 mg/m^2^) every two weeks [28]. Among the 5 NF1 patients, two showed partial radiological responses, and three achieved stable disease, with none exhibiting disease progression during BBT. Clinically, NF1 patients demonstrated improvements or stability, particularly in vision, where deterioration was halted or reversed. Across all patients, 69% achieved stable disease, and 19% demonstrated partial radiological responses. Toxicities were manageable, with irinotecan discontinued in some cases due to gastrointestinal side effects. No severe toxicities related to bevacizumab were reported [28].

The findings suggest that bevacizumab-based therapy is effective and well-tolerated in NF1 patients with recurrent LGG, providing disease control and functional preservation, including vision stabilization or improvement.

A nationwide evaluation included 88 children with pediatric LGG treated across 11 centers in the UK between 2009 and 2020, of whom 24% had NF1. Most tumors were OPGs, with the majority involving the chiasm or post-chiasmatic regions. Bevacizumab was primarily administered with irinotecan as a third-line therapy, although earlier use in selected cases was reported. Clinical and radiological responses were evaluated using standardized criteria, with a particular focus on visual acuity outcomes.

Results demonstrated that BBT achieved disease control during treatment in 88% of cases, with partial radiological responses in 40% and stable disease in 49%. Visual outcomes were particularly favorable in NF1-associated OPGs, with superior visual EFS compared to non-NF1 cases (*p* = 0.023) [29]. Among all patients, 29% experienced improvement in VA, 49% showed stability, and 22% had deterioration. While the median radiological progression-free survival (PFS) was 17 months, visual preservation appeared more durable, highlighting a potential role for BBT in protecting functional vision. The findings underscore the importance of timely intervention with BBT in progressive OPGs to optimize visual preservation and quality of life, emphasizing the need for multidisciplinary management and individualized treatment approaches [29].

### 3.3. Non-Optical Gliomas

**Recommendation 7**. It is recommended for pediatric patients with NF1, and gliomas not associated with symptomatic or progressive optic pathway, the complete surgical excision whenever possible. When inoperable, chemotherapy should be instituted, always avoiding the association with radiotherapy. (100% agreement in the first round)—Evidence IV.

No articles supporting this evidence were found. This recommendation is based on expert opinion.

**Recommendation 8**. Pediatric patients with NF1 who have symptomatic gliomas—with or without optic pathway involvement—should be referred early to specialized cancer or neurological treatment centers. (100% agreement in the first round)—Evidence IV.

No articles supporting this evidence were found. This recommendation is based on expert opinion.

### 3.4. Plexiform Neurofibromas

**Recommendation 9.** It is recommended that pediatric patients with NF1 and asymptomatic plexiform neurofibromas undergo periodic clinical and radiological (MRI) surveillance. (93.75% agreement in the first round)—Evidence IIA.

Plexiform neurofibromas (PN) are common in patients with NF1, can grow significantly during childhood, and carry a risk of malignant transformation, primarily to MPNST in adults. While surgery or mitogen-activated extracellular signal-regulated kinase (MEK) inhibitors are not indicated for asymptomatic PN, routine surveillance is considered crucial due to their growth potential and risk of malignancy. A study published in 2024 aimed to determine whether children with NF1 who show no PN on initial whole-body MRI (WB-MRI) can develop these tumors later in childhood or adolescence [30]. Researchers retrospectively reviewed WB-MRI scans of 17 pediatric NF1 patients (median initial age: 9 years) who had no PN at baseline and were followed for a median of 9 years. Two out of 17 children developed new PN during this follow-up; both tumors were identified between the ages of 10 and 16. The majority (15/17) showed no development of distinct tumors over at least 6 years [30].

The findings indicate that even in the absence of initial tumor burden, pediatric NF1 patients can develop PN during adolescence. As a result, the study supports the recommendation that a second WB-MRI should be performed at the transition to adulthood, especially if the initial MRI occurred significantly before age 18, to monitor for new tumor development and potential complications [30].

**Recommendation 10**. It is recommended that pediatric patients with NF1 and symptomatic or disfiguring plexiform neurofibromas be evaluated by a multidisciplinary team. Where possible and safe, partial or complete surgical resection of the lesion should be considered. (90.91% agreement in the second round)—Evidence III.

In a retrospective study conducted by Collins-Sawaragi et al. involving 127 children with NF1, the authors reported disfigurement in 57% (72/127) of patients with plexiform neurofibroma. Impairment of function was seen in 23% (29/127) and included motor difficulties, upper airway compression, spinal cord compression.

Indications for surgery were disfigurement in 23/35 patients, threat to function (brain herniation) in 1 patient, and other symptoms from PN in 11 patients (symptomatic cord compression/myelomalacia in 2 patients; MPNST in 2 patients; pain, discomfort, or enlargement in 5 patients; restricted neck movement in 1 patient; difficulty in defecation in 1 patient). Surgery performed was debulking in 28/35, total excision in 5/35, wide resection in 1/35, while one patient did not proceed to surgery. All disfigurement symptoms improved with surgery, with reasonable patient satisfaction and no complications, although some patients have subsequently required repeat operations as expected for craniofacial PN surgery. The 11 patients undergoing surgery for symptoms of PN all improved, with only one regrowth of PN due to MPNST [31]. In a longitudinal cohort study involving a review of medical charts of 106 pediatric and 461 adult patients with NF1 and PN, the authors observed that pain was the most frequently documented symptom among both pediatric and adult patients. A requirement for surgery was reported for 15 of 40 PN (38%) in the 34 pediatric patients and for 86 of 191 PN (45%) in the 159 adult patients with large PN. Among the large PN that required surgery, most had required one procedure (12 of 15 PN [80%] in pediatric patients and 54 of 86 PN [62%] in adult patients) at the time of data cut-off. Procedures included complete resection (seven of 15 procedures [47%] in pediatric patients and 34 of 86 procedures [40%] in adult patients) and debulking (eight of 15 procedures [53%] in pediatric patients and 52 of 86 procedures [60%] in adult patients) [32].

**Recommendation 11**. For pediatric patients with NF1 and symptomatic, inoperable plexiform neurofibromas, treatment with MEK inhibitors is recommended as the best therapeutic option to reduce tumor volume, alleviate pain, and improve quality of life and functional outcomes. (93.75% agreement in the first round)—Evidence IIA.

A systematic review published in 2021 identified four treatments, as reported in 9 articles, that can be used in patients with inoperable PN. Selumetinib has shown an overall response rate of 68% in children with NF1 and symptomatic inoperable PNs and was associated with pain improvement and a manageable adverse events profile. This led to the Food and Drug Administration (FDA) approval of selumetinib in May 2020 [33].

Gross et al. (2020) conducted a Phase 2 clinical trial to understand the response rate among patients with plexiform neurofibromas treated with selumetinib. Fifty children were enrolled. After 1 year of treatment, the mean decrease in child-reported tumor pain-intensity scores was 2 points, considered a clinically meaningful improvement. In addition, clinically meaningful improvements were seen in child-reported and parent-reported interference of pain in daily functioning (38% and 50%, respectively) and overall health-related quality of life (48% and 58%, respectively) as well as in functional outcomes of strength (56% of patients) and range of motion (38% of patients). The authors concluded that most children with NF1 and inoperable plexiform neurofibromas experienced durable tumor shrinkage and clinical benefit from selumetinib [34].

A recent publication reported a multicenter, open-label phase IIb trial with adults and children. In the ReNeu trial, treatment of children with neurofibromatosis type 1-associated plexiform neurofibromas (NF1-PN) using mirdametinib showed promising results: 52% of children achieved a confirmed objective tumor response (defined as at least a 20% reduction in target tumor volume on MRI), with a median best reduction in tumor volume of 42%. Responses were durable, with 76% of responders maintaining their response for at least 12 months. Additionally, children experienced significant and sustained improvements in patient or parent proxy-reported measures of worst tumor pain, pain interference, and health-related quality of life, beginning early in treatment and continuing throughout the study period. The most common treatment-related adverse events in children were dermatitis acneiform, diarrhea, and paronychia, and the safety profile was generally manageable, with only 9% of children discontinuing due to adverse events. Mirdametinib therefore demonstrates meaningful efficacy, tolerability, and quality-of-life benefits for children with symptomatic, inoperable NF1-PN [35]. Other MEK inhibitors, such as trametinib and binimetinib (children), have also demonstrated preliminary results [36].

In a single-arm phase II open-label trial that included 90 patients, both children (n = 60) and adults (n = 30) received selumetinib for 4 to 26 cycles. The most common adverse events (AEs) were paronychia (n = 62, 14.7%), followed by acneiform rash (n = 61, 14.5%) and skin infection (n = 59, 14.0%). Except for 20 (4.7%) cases of Common terminology criteria for adverse events (CTCAE) grade 2 AEs, all other AEs were grade 1. Eighty-eight patients (97.8%) out of 90 patients in the intention-to-treat group showed a median re duction of 40.8% (range, 4.2–92.2%) in the PN volume. For the per-protocol analysis, which included 89 patients, the response rate was 98.9% (88 out of 89 patients). The verbal Comprehension Index (VCI), Perceptual Reasoning Index (PRI), working memory index, Processing Speed Index (PSI), and Full-Scale Intelligence Quotient (FSIQ) were measured. The improvements were statistically significant for VCI and FSIQ (group 1), and PSI (groups 1 and 3). The proportion of patients with borderline (FSIQ; 70~79) or overt intellectual disability (FSIQ < 70) in terms of VCI (children), PRI (children and adults), PSI (children and adults), and FSIQ (children and adults) decreased because of this improvement. After receiving selumetinib, 63.6% of children and 78.6% of their parents reported an improvement in their QoL [37].

In another study, the authors observed tumor reduction in 16 out of 17 plexiform neurofibromas (94%), while only one tumor remained stable, and none grew during the follow-up period. The media size reduction was 23%. The greatest radiological response was a 57% reduction, while the smallest, being the only case of tumor stabilization, was 14% [38]. This case series suggests that, given the poor results of the surgical approach, the initiation of selumetinib should not be delayed or discontinued in patients with severe symptomatic PN, as these can cause functional impairment due to the local growth of the masses, as well as significant symptoms and a poor quality of life. Despite these positive results, however, this report has some limitations. The manual radiological measurement of the PN, the lack of randomization, the small size of this cohort, and the limited follow-up in some individuals prevent the possibility of drawing definitive conclusions regarding the tolerability and efficacy of selumetinib in children [38].

Coltin H et al. reported a case series of 19 patients in which selumetinib treatment started at a mean age of 10.7 years (range 2.5–18). Reasons for treatment included pain (14/19), PN progression (10/19), motor dysfunction (9/19), disfigurement (6/19), respiratory compromise (4/19), renal/bladder dysfunction (3/19), scoliosis (2/19), and edema (1/19). Most PNs were multifocal. Maintenance doses ranged from 1 to 1.6mg/kg/day or 40 to 50 mg/m^2^/day, divided twice daily. Symptoms improved or stabilized in 73.7% and 26.3% of patients, respectively, without any reports of worsening. Of the 14 patients with pain, selumetinib was associated with improvement or resolution in 13. All PNs were grossly stable on MRI during treatment. Compared to trametinib, selumetinib was associated with superior (4/8) or comparable (4/8) symptom control; and with halted tumor growth (2/8) or ongoing stability (6/8). It was considered safe, as most of the adverse events were grade 1 [39].

Dombi E et al., evaluating 24 patients in a phase I trial receiving selumetinib at three dose levels, observed a decrease from baseline in PN volume in all patients (median change, −31%; range, −5.8 to −47). The maximum response to selumetinib was achieved after a median of 20 cycles (range, 5–42). Partial responses were durable, in that they were sustained for a median of 23 cycles (range, 6 to 42), and 15 of the 17 patients with partial response maintain their response status to date. These early-phase data suggested that children with NF1 and inoperable PN benefited from selumetinib without experiencing excessive toxic effects [40].

Gross et al. analyzed the follow-up of a Phase 1 and 2 study involving 74 patients (24 in Phase 1 and 50 in Phase 2). They showed that most children with NF1-related PN experienced lasting tumor reduction and continued pain improvement beyond what was previously reported at 1 year, with no new safety signals identified [41].

**Recommendation 12**. Rigorous monitoring is recommended for all pediatric patients with NF1 and symptomatic, inoperable plexiform neurofibromas who are treated with MEK inhibitors, to ensure appropriate management of side effects and optimization of dosing. (100% agreement in the first round)—Evidence IIA.

In a systematic review and meta-analysis of 126 patients using selumetinib, the authors identified diarrhea, with a combined AE rate of 63.8% (95% CI 52.9–73.4%; I^2^ = 0%), and increases in creatine kinase (CK) levels, with a combined AE rate of 63.3% (95% CI 35.6–84.3%; I^2^ = 75%), as the main adverse events [33].

An open-label phase I study in Japan that included 12 pediatric patients showed that all patients had baseline PN-related morbidities, most commonly disfigurement (91.7%), pain (58.3%), and motor weakness. Most frequently reported any-grade adverse events were dermatologic and gastrointestinal. Two patients (16.7%) had their dose interrupted and then decreased because of AEs (grade 2 ejection fraction decrease); both patients were able to continue selumetinib treatment at a lower dose after ejection fraction normalization (one patient) or improvement (one patient). No patients reported worsening of PN-related morbidities [42].

In another study with 90 patients, the most common AE was paronychia (n = 62, 14.7%), followed by acneiform rash (n = 61, 14.5%) and skin infection (n = 59, 14.0%). Except for 20 (4.7%) cases of CTCAE grade 2 AEs, all other AEs were grade 1. In a Portuguese real-world case series studying 19 inoperable PN patients, treatment was discontinued in one patient after 168 days on selumetinib due to the lack of clear clinical benefit and a drop in left ventricular ejection fraction, which fully resolved within a month after stopping the treatment. The remaining 18 patients exhibited clinical improvement, primarily within the first 60–90 days. Grade 2 AE were acneiform rash (n = 7), asymptomatic left ventricular ejection fraction reduction (n = 4), paronychia (n = 3), nausea and vomiting (n = 1), erythematous rash (n = 1), and neutrophil count decrease (n = 1). Grade 3 AE occurred in two patients (asymptomatic CPK increase) [43].

In another study, nine patients treated with selumetinib participated in the INSPECT study; the authors reported that 18 patients taking MEK inhibitors experienced treatment discontinuation in 5 of 18 cases. The reasons for stopping the MEK inhibitor included side effects in one patient, lack of clinical benefit in two, and both side effects and a lack of clinical benefit in two additional patients. Side effects were categorized as cutaneous (8 out of 18 mild, 5 out of 18 moderate, 5 out of 18 significant), gastrointestinal in 8 out of 18 (2 out of 18 mild, 2 out of 18 moderate, 4 out of 18 significant), along with 1 case of gross hematuria and 1 case of weight gain [31].

These data collectively indicate that adverse events are common and that close monitoring of all pediatric patients taking MEK inhibitors is necessary.

**Recommendation 13**. Whenever possible, a whole-body MRI is recommended for all patients with NF1 during the transition to adulthood, regardless of whether plexiform neurofibromas have been previously identified. (87.50% agreement in the first round)—Evidence III.

Although few studies have focused on this topic, some have demonstrated benefits specifically for the diagnosis of internal plexiform neurofibromas in adolescents and young adults. A retrospective study found that the majority of the pediatric NF1 patients (15 of 17 children) without initial tumor burden did not develop any distinct tumors on follow-up MRI scans for at least 6 years. In two out of 17 children without an initial tumor burden, new PN were identified on follow-up MRI scans. One of the PN growth rates across approximately 7 years showed that the growth occurred during adolescence [30]. In the ERN GENTURIS consensus, there is a recommendation that pediatric patients with neurofibromatosis type 1 (NF1) undergo at least one whole-body magnetic resonance imaging (WB-MRI) scan during the transition from adolescence to adulthood, even if no tumors were detected in earlier childhood imaging. This approach is advised because PN can develop or grow during adolescence, and early detection is essential due to the risk of significant tumor growth and potential malignancy in adulthood. By performing WB-MRI during this critical transition period, clinicians can identify newly developed or asymptomatic tumors, better assess tumor burden, and guide further surveillance or intervention when necessary [20].

### 3.5. Malignant Peripheral Nerve Sheath Tumors (MPNST)

**Recommendation 14**. Pediatric patients with NF1 and plexiform neurofibromas should be closely monitored for signs of malignant transformation, including persistent pain or change in pre-existing pain pattern, increased tumor growth velocity, and/or neurological deficit. (100% agreement in the first round)—Evidence III.

People with NF1 are predisposed to developing both benign and malignant tumors [44]. Among malignant tumors, MPNSTs are particularly concerning due to their incidence and poor prognosis when diagnosed at advanced stages [45,46].

The lifetime risk of developing MPNST in individuals with NF1 is estimated at 8–13%, representing a 9000-fold increase compared to the general population, which equates to an annual risk of approximately 0.16% [47,48,49,50]. In addition, MPNSTs are also one of the leading causes of death in individuals with NF1 [49]. Malignant progression in NF1 is often marked by persistent pain, neurological deficits, or rapid tumor enlargement, typically originating from pre-existing plexiform neurofibromas [45,51]. In a cohort reported by Friedrich et al., all patients with MPNST were symptomatic [51]. Similarly, in a single-institution retrospective analysis, the most common initial symptoms were pain (55%) and palpable masses (45%) [52]. Clinical data from Miao et al. revealed that among 77 NF1 patients with MPNST, 64.9% presented with a palpable mass, 61% reported pain, and 20.8% exhibited neurological symptoms [53]. Valentin et al. reported comparable findings in their study of 131 NF1 patients, with pain and palpable masses as the most common presenting symptoms (50% each), followed by neurological deficits (20%) [54]. Another retrospective analysis of NF1 patients identified clinical variables associated with MPNST, including pain (positive predictive value [PPV] 67%, negative predictive value [NPV] 75%), neurological symptoms (PPV 57%, NPV 91%), and tumor enlargement (PPV 95%, NPV 92%) [55].

Although pain, tumor enlargement, and neurological symptoms are commonly observed, these signs are not always specific to malignant transformation. In a large 20-year study investigating the incidence of MPNSTs in individuals with NF1, approximately one-third of patients who developed an MPNST lacked outward physical signs or symptoms of PN [45]. Similarly, Azizi et al. reported cases of asymptomatic patients with malignant lesions, where early detection enabled safe surgical removal [56].

Given the aggressive nature of MPNSTs, comprehensive and detailed clinical assessments are crucial. Children may have difficulty articulating symptoms such as pain, tumor growth, or neurological deficits as clearly as adults, making thorough investigation of any reported complaints essential. This emphasizes the critical importance of vigilant clinical monitoring to facilitate early detection of MPNSTs and improve outcomes.

**Recommendation 15.** Pediatric patients with NF1 and plexiform neurofibromas showing possible signs of malignancy should undergo MRI and a thorough clinical assessment before deciding if a biopsy is necessary. (93.75% agreement in the first round)—Evidence III.

Distinguishing between benign and malignant peripheral nerve sheath tumors based solely on clinical symptoms is challenging, as both can present with similar symptoms [57]. Non-invasive methods to distinguish benign symptomatic neurofibromas from those undergoing malignant transformation remain a significant clinical hurdle [58]. While conventional MRI and CT scans provide detailed anatomical insights and tumor growth progression, they lack the precision to reliably differentiate malignant from benign lesions, especially in cases of MPNSTs. In adult NF1 patients, 18F-FDG PET/CT has emerged as a valuable diagnostic tool, utilizing standardized uptake values (SUVs) and tumor-to-liver ratios as semi-quantitative markers for characterizing MPNSTs. Compared to whole-body volumetric MRI, 18F-FDG PET/CT demonstrates significantly higher sensitivity for detecting MPNSTs in symptomatic NF1-associated lesions (100% vs. 67%) [59]. However, data on its application in pediatric patients remain limited, based on small, retrospective studies [59,60].

The use of 18F-FDG PET/CT to detect premalignant or malignant lesions in children presents challenges, such as the risk of overdiagnosis, high costs, and radiation exposure [44]. Additionally, the cumulative radiation exposure from serial imaging in children with cancer predisposition syndromes like NF1 must be minimized, given the heightened risk of secondary radiation-induced malignancies and vasculopathies. Surveillance imaging in children under 10 years of age must balance the benefits against potential risks. Sedation is often required for imaging in younger children, adding another layer of complexity [61].

**Recommendation 16.** Imaging-guided percutaneous biopsy (PET/CT, PET MRI, or US) is currently the most effective approach for the diagnosis of MPNST in pediatric patients with NF1 and plexiform neurofibromas with suspected malignancy. (87.50% agreement in the first round)—Evidence III.

Currently, there is no established consensus on the optimal diagnostic strategy for early detection of MPNST in NF1 patients. While PET CT imaging alone lacks specificity for diagnosing NF1-related MPNST, histopathological analysis remains essential for accurate diagnosis and treatment planning. A retrospective study by Brahmi et al. with adolescents and adults with NF-1 demonstrated the value of PET/CT-guided percutaneous biopsies, achieving a diagnostic accuracy rate of 96% (25/26) with sensitivity, specificity, PPV, and NPV of 94%, 100%, 100%, and 89%, respectively. These biopsies, performed in an outpatient setting with minimal complications, proved to be safe and effective. However, one false negative occurred due to the tumor’s heterogeneity and inflammatory components, highlighting the need for multiple biopsies in larger tumors [57]. This technique should be conducted under the guidance of a multidisciplinary team to mitigate the risk of false negatives and ensure comprehensive tumor evaluation [57].

**Recommendation 17.** Surgical resection with wide, tumor-free margins is the cornerstone of treatment for malignant peripheral nerve sheath tumors (MPNST) associated with NF1 and is considered the therapy of choice. (87.50% agreement in the first round)—Evidence III.

Similarly to the diagnostic strategy, the management of MPNST lacks standardized guidelines, especially for pediatric patients. The lack of consensus on treatment protocols results from the limited number of studies with small cohort sizes. While treatment options for adult MPNST are more established, the management of pediatric MPNST remains less clearly defined [62]. The primary treatment for MPNST is surgical excision, which is strongly associated with improved survival outcomes. The goal is to achieve wide surgical margins, as this minimizes local recurrence rates and enhances survival prospects [63]. A prospective European study by Noesel et al. demonstrated that resection of small tumors was effective for both NF1 and non-NF1 patients. However, NF1 patients often present with larger tumors and continue to have poorer outcomes [64]. Notably, the majority of pediatric cases of MPNSTs are initially unresectable at diagnosis [64].

**Recommendation 18.** In cases of unresectable malignant peripheral nerve sheath tumors (MPNST), with partial or large resection (>5 cm), and in the absence of access to target therapy, it is recommended to consider the inclusion of radiotherapy and/or chemotherapy in the therapeutic plan. The decision should be individualized based on tumor location and extent. (86.7% agreement in the second round)—Evidence III.

For unresectable or large, localized pediatric MPNSTs, radiotherapy and chemotherapy are often included in treatment plans. However, their roles remain controversial due to limited evidence of effectiveness. A study by Martin et al., using data from the Netherlands Cancer Registry (NCR) and the Dutch Pathology Database (PALGA) from 1989 to 2017, found that among pediatric NF1 patients with MPNST, surgical removal was performed in 84.6% of cases, with complete excision achieved in 57.1% of cases. Preoperative radiotherapy was given in 11.5% of cases, while postoperative radiotherapy was used in 34.6%. Chemotherapy was administered in 26.9% of cases, with preoperative, postoperative, and chemotherapy-only regimens used in 11.5%, 11.5%, and 3.8%, respectively [65]. Radiotherapy is primarily employed to enhance local control, although it has not been shown to improve overall prognosis. A study by Meister et al. found that radiotherapy did not significantly improve local control in NF1 patients, with local failure rates of 9/11 in patients receiving radiotherapy and 13/27 in those without radiotherapy (*p* = 0.056) [66]. In pediatric cases, efforts are made to limit radiation doses to reduce side effects, such as growth impairment in irradiated tissues. However, these reduced doses lack support from randomized trials and may compromise the effectiveness of treatment.

**Recommendation 19**. If chemotherapy is indicated, the first-line treatment for pediatric patients with NF1 and malignant peripheral nerve sheath tumors (MPNST) is recommended to be a combination of ifosfamide and doxorubicin. However, this decision can be individualized based on tumor characteristics, the patient’s clinical condition, and the experience of the multidisciplinary team. (86.7% agreement in the second round)—Evidence III.

The role of chemotherapy in MPNST, particularly in cases associated with NF1, remains poorly defined. It is primarily used in advanced or unresectable disease, where achieving negative margins through surgery alone is unlikely. Standard chemotherapy regimens include combinations of vincristine, cyclophosphamide, ifosfamide, doxorubicin, and dactinomycin. However, chemotherapy response rates are generally lower in NF1 patients. A study by Carli et al., evaluating 167 untreated pediatric patients enrolled in Italian and German studies between 1975 and 1998, found that 17% of the patients had NF1. The study reported a significantly lower chemotherapy response rate in NF1 patients (17.6%; 3 of 17) compared to non-NF1 patients (55.3%; 26 of 47; *p* = 0.007). NF1 patients also had poorer outcomes, with a 5-year OS of 32% and PFS of 19%, potentially due to factors such as unfavorable clinical presentation and reduced chemotherapy sensitivity [67]. In contrast, Meister et al. found no significant difference in chemotherapy response rates (complete or partial response) between NF1 and non-NF1 patients (38% vs. 41%, respectively; *p* = 0.87). However, most NF1 patients who received radiotherapy also received chemotherapy, making it difficult to assess the independent effects of each treatment modality [66].

Given the poor prognosis of NF1-associated MPNST, more aggressive therapeutic regimens may be necessary. Combining chemotherapy and radiotherapy may provide a potential way to improve outcomes in NF1 patients, although study results are still debated, and the risk of secondary malignancies from radiotherapy must be carefully evaluated. This highlights the need for new therapeutic strategies to enhance the prognosis of MPNST in NF1 patients.

### 3.6. Melanoma

**Recommendation 20.** Currently, there is insufficient data in the literature, particularly in the pediatric population, to provide formal recommendations regarding melanoma development in patients with NF1. However, annual follow-up with a dermatologist and an ophthalmologist is suggested, although the optimal age to begin this surveillance remains unclear. (100% agreement in the first round)—Evidence IV.

No articles supporting this evidence were found. This recommendation is based on expert opinion.

### 3.7. Juvenile Myelomonocytic Leukemia (JMML)

**Recommendation 21**. Although rare, juvenile myelomonocytic leukemia (JMML) has been reported in pediatric patients with NF1. Healthcare professionals should be aware of clinical signs such as adenomegaly, hepatosplenomegaly, and pallor, but extensive investigations should be avoided unless clearly indicated. (100% agreement in the first round)—Evidence III.

Juvenile myelomonocytic leukemia (JMML) is a rare and aggressive childhood cancer associated with mutations in the RAS/MAPK signaling pathway, including NF1 mutations, found in 22% of cases according to a recent study using a 51gene myeloid panel [68]. While the overall risk of JMML in NF1 patients is low, NF1 mutations are implicated in some cases, emphasizing the importance of genomic profiling for treatment decisions, such as hematopoietic cell transplantation. However, routine JMML screening for NF1 patients is not necessary [69].

### 3.8. Pheochromocytomand Paraganglioma

**Recommendation 22.** Laboratory and imaging screening is not recommended for the investigation of pheochromocytoma and paraganglioma in pediatric patients with asymptomatic NF1. (100% agreement in the first round)—Evidence III.

Laboratory testing should be undertaken once there is clinical suspicion of a pheochromocytoma (PCC) and paraganglioma (PGL). Measurements of plasma and 24 h urinary catecholamines (epinephrine, norepinephrine, and dopamine) and urinary vanillylmandelic acid (VMA) have fallen out of favor due to their lower sensitivity and specificity; assessing catecholamine metabolites is now recommended. These include plasma-free metanephrines (metanephrine and normetanephrine) and 24 h urinary fractionated metanephrines [70].

**Recommendation 23**. For pediatric patients with NF1 who present with symptoms such as hypertension, headache, or palpitations, diagnostic evaluation for pheochromocytoma (PCC) and paraganglioma (PGL) is recommended, including biochemical testing (catecholamines and urinary or plasma metanephrines) and imaging studies. (100% agreement in the first round)—Evidence III.

Bhola and Bunchman published a review in 2017. They attested that the clinical presentation of PGL and PCC varies, with sustained hypertension observed in 60–90% of pediatric cases. Symptomatology may depend on the type of hormone being secreted. Individuals with epinephrine-secreting tumors can present with hypoglycemia and hypotensive shock due to excess catecholamine production and circulatory collapse. Dopamine-secreting tumors are usually asymptomatic, delaying diagnosis until the tumor’s mass effect becomes apparent. The mass effect from non-functional head and neck paragangliomas can cause dysphagia, hoarseness, hearing disturbances, and pain [70]. As mentioned previously, laboratory tests should only be performed if there is clinical suspicion of PCC or PGL [70].

### 3.9. Gastrointestinal Stromal Tumors (GIST)

**Recommendation 24.** Although rare, gastrointestinal stromal tumors (GISTs) have been reported in pediatric patients with NF1. Healthcare professionals should be alert to signs and symptoms such as gastrointestinal discomfort, weight loss, anemia, gastrointestinal bleeding, abdominal pain, a moderately palpable abdominal mass, or intestinal obstruction. Diagnostic evaluation for GISTs should be performed only when there is clinical suspicion. (100% agreement in the first round)—Evidence IV

Currently, there is not enough literature to support this recommendation. This recommendation is based on expert opinion. According to the ERN GENTURIS consensus, healthcare professionals should remain alert for signs and symptoms suggestive of GISTs, including gastrointestinal discomfort, weight loss, anemia, gastrointestinal bleeding, abdominal pain, a palpable abdominal mass, or intestinal obstruction. Notably, diagnostic tests for GISTs should only be performed if there is clinical suspicion—routine screening in asymptomatic individuals is not recommended. This targeted strategy strikes a balance between the need for early detection and the goal of avoiding unnecessary procedures in the pediatric NF1 population [20].

## 4. Conclusions

This consensus represents the first Brazilian recommendations on malignant and benign tumors in pediatric patients with NF1, providing a framework to standardize and optimize clinical management of this disease. Although our article does not possess the excellence and robustness of the ERN GENTURIS tumor surveillance guidelines for individuals with neurofibromatosis type 1, we recognize that both studies aim to improve practices among specialists in the field and increase awareness of NF1 among healthcare professionals and the general public. This awareness promotes early diagnosis and intervention. Our work focuses on guiding widely accessible examinations and care within the national health system, specifically avoiding recommendations for examinations and procedures that are not available to the general population.

Finally, most recommendations reached consensus in the first round of discussion. While the recommendations received strong agreement, some are based on studies with notable limitations, highlighting the need for refinement as new evidence emerges. The findings emphasize the importance of robust, multicenter research to validate these methods and broaden their applicability across diverse patient populations, ensuring ongoing improvements in clinical outcomes.

## Figures and Tables

**Table 1:** Search strategies used in the MEDLINE (via PubMed), LILACS (via BVS), and Cochrane databases.

Database	Terms	Results
MEDLINE (PubMed)	((“Pediatrics”[MeSH Terms] OR “Child”[MeSH Terms] OR “Adolescent”[MeSH Terms] OR “teenager*”[Title/Abstract] OR “kids”[Title/Abstract] OR “children”[Title/Abstract]) AND (“neurofibromatosis 1”[MeSH Terms] OR “neurofibromatosis type 1”[Title/Abstract] OR “NF1”[Title/Abstract])) AND (y_10[Filter])	1846
LILACS (via BVS)	(Pediatrics OR Child OR Adolescent OR teenager* OR kids OR children) AND (neurofibromatosis 1 OR neurofibromatosis type 1 OR NF1)	42
Cochrane Library	(Pediatrics OR Child OR Adolescent OR teenager* OR kids OR children):ti,ab,kw AND (neurofibromatosis 1 OR neurofibromatosis type 1 OR NF1):ti,ab,kw with Cochrane Library publication date from Jan 2014 to Nov 2024	125

Legend: Search made on 31 January 2025.

**Table 2:** Number of experts invited for each round.

Links Sent According to the Area of Expertise	Number of Experts Invited in the First Round	Number of Experts Invited in the Second Round
Link 1—Related to Glioma recommendations	22	12
Link 2—Related to Plexiform Neurofibromas and Malignant Peripheral Nerve Sheath Tumors	24	16
Link 3—Related to all other categories	19	Consensus reached in the first round
TOTAL	65 *	28 *

* It is important to note that 26 experts responded in the first round. Some were invited to respond to more than one link.

**Table 3:** Classification of level of evidence [5].

Evidence Level Description	
IA	Evidence from meta-analysis of RCTs
IB	Evidence from at least 1 RCT or meta-analysis using RCTs and non-RCTs
IIA	Evidence from at least 1 controlled study without randomization
IIB	Evidence from at least 1 other type of quasi-experimental study
III	Evidence from non-experimental descriptive studies, such as comparative studies, correlation studies, and case–control studies
IV	Evidence from expert committee reports or opinions, or clinical experience of respected authorities, or both

Adapted from Shekelle et al. [5]: RCT; Randomized Controlled Trial.

**Table 4:** Recommendations, percentage, and the level of evidence.

N	Recommendations	Consensus Percentage	Level of Evidence
1	It is recommended for all patients newly diagnosed with NF1 should be followed up by an ophthalmologist to evaluate visual acuity, visual fields, pupillary reflexes, eye movements, and optic disc aspect. Ophthalmic follow-up should be annual until early adulthood.	100%	III
2	It is recommended that pediatric patients with NF1 undergo regular clinical ophthalmologic evaluations, complemented by imaging tests such as optical coherence tomography (OCT), to investigate choroidal and retinal alterations. The evaluation should be performed at least once, according to the clinical and visual evolution of the patient.	100%	III
3	Routine screening with MRI of the central nervous system and optical pathways is recommended for pediatric patients with NF1 and associated symptoms such as recent visual impairment or physical signs such as proptosis, strabismus, nystagmus, persistent headache, precocious puberty, abnormal growth patterns, or other ophthalmological and/or neurological signs and symptoms suggestive of optic tumors. In addition, in regions with limited access to specialized ophthalmologic evaluation, MRI in pediatric patients with asymptomatic NF1 represents an important and safe tool for monitoring these patients.	100%	III
4	Biopsy is not recommended for diagnostic confirmation of optic pathway gliomas in pediatric patients with NF1.	100%	III
5	An initial approach with vincristine and carboplatin is recommended as the first line of treatment for pediatric patients with NF1 and the presence of symptomatic optic pathway gliomas.	83%	III
6	The following therapeutic options are recommended as second-line therapy for pediatric patients with NF1 and the presence of recurrent or refractory optic pathway gliomas: vinblastine, carboplatin, vinorelbine, or bevacizumab, isolated or in combination.	83%	III
7	It is recommended for pediatric patients with NF1, and gliomas not associated with symptomatic or progressive optic pathway, the complete surgical excision whenever possible. When inoperable, chemotherapy should be instituted, always avoiding the association with radiotherapy.	100%	IV
8	Pediatric patients with NF1 who have symptomatic gliomas—with or without optic pathway involvement—should be referred early to specialized cancer or neurological treatment centers.	100%	IV
9	It is recommended that pediatric patients with NF1 and asymptomatic plexiform neurofibromas undergo periodic clinical and radiological (MRI) surveillance.	93.75%	IIA
10	It is recommended that pediatric patients with NF1 and symptomatic or disfiguring plexiform neurofibromas be evaluated by a multidisciplinary team. Where possible and safe, partial or complete surgical resection of the lesion should be considered.	90.91	III
11	For pediatric patients with NF1 and symptomatic, inoperable plexiform neurofibromas, treatment with MEK inhibitors is recommended as the best therapeutic option to reduce tumor volume, alleviate pain, and improve quality of life and functional outcomes.	93.75	IIA
12	Rigorous monitoring is recommended for all pediatric patients with NF1 and symptomatic, inoperable plexiform neurofibromas who are treated with MEK inhibitors, to ensure appropriate management of side effects and optimization of dosing.	100%	IIA
13	Whenever possible, a whole-body MRI is recommended for all patients with NF1 during the transition to adulthood, regardless of whether plexiform neurofibromas have been previously identified.	87.50%	III
14	Pediatric patients with NF1 and plexiform neurofibromas should be closely monitored for signs of malignant transformation, including persistent pain or change in pre-existing pain pattern, increased tumor growth velocity, and/or neurological deficit.	100%	III
15	Pediatric patients with NF1 and plexiform neurofibromas showing possible signs of malignancy should undergo MRI and a thorough clinical assessment before deciding if a biopsy is necessary.	93.75%	III
16	Imaging-guided percutaneous biopsy (PET/CT, PET MRI, or US) is currently the most effective approach for the diagnosis of MPNST in pediatric patients with NF1 and plexiform neurofibromas with suspected malignancy.	87.50%	III
17	Surgical resection with wide, tumor-free margins is the cornerstone of treatment for malignant peripheral nerve sheath tumors (MPNST) associated with NF1 and is considered the therapy of choice.	87.50%	III
18	In cases of unresectable malignant peripheral nerve sheath tumors (MPNST), with partial or large resection (>5 cm), and in the absence of access to target therapy, it is recommended to consider the inclusion of radiotherapy and/or chemotherapy in the therapeutic plan. The decision should be individualized based on tumor location and extent.	86.70%	III
19	If chemotherapy is indicated, the first-line treatment for pediatric patients with NF1 and malignant peripheral nerve sheath tumors (MPNST) is recommended to be a combination of ifosfamide and doxorubicin. However, this decision can be individualized based on tumor characteristics, the patient’s clinical condition, and the experience of the multidisciplinary team.	86.70%	III
20	Currently, there is insufficient data in the literature, particularly in the pediatric population, to provide formal recommendations regarding melanoma development in patients with NF1. However, annual follow-up with a dermatologist and an ophthalmologist is suggested, although the optimal age to begin this surveillance remains unclear.	100%	IV
21	Although rare, juvenile myelomonocytic leukemia (JMML) has been reported in pediatric patients with NF1. Healthcare professionals should be aware of clinical signs such as adenomegaly, hepatosplenomegaly, and pallor, but extensive investigations should be avoided unless clearly indicated.	100%	III
22	Laboratory and imaging screening is not recommended for the investigation of pheochromocytoma and paraganglioma in pediatric patients with asymptomatic NF1.	100%	III
23	For pediatric patients with NF1 who present with symptoms such as hypertension, headache, or palpitations, diagnostic evaluation for pheochromocytoma (PCC) and paraganglioma (PGL) is recommended, including biochemical testing (catecholamines and urinary or plasma metanephrines) and imaging studies.	100%	III
24	Although rare, gastrointestinal stromal tumors (GISTs) have been reported in pediatric patients with NF1. Healthcare professionals should be alert to signs and symptoms such as gastrointestinal discomfort, weight loss, anemia, gastrointestinal bleeding, abdominal pain, a moderately palpable abdominal mass, or intestinal obstruction. Diagnostic evaluation for GISTs should be performed only when there is clinical suspicion.	100%	IV

## Data Availability

Data available upon request: Data can be provided upon request to the corresponding author.

## Abbreviations

The following abbreviations are used in this manuscript:

AE	Adverse Event
adjOR	Adjusted odds ratio
BBT	Bevacizumab-based therapy
CK	Creatine kinase
CTCAE	Common terminology criteria for adverse events
CV	Carboplatin and vincristine
cpRNFL	Circumpapillary retinal nerve fiber layer
EFS	Event-free survival
FDA	Food and Drug Administration
FSIQ	Full-Scale Intelligence Quotient
GBCA	Gadolinium-based contrast agents
GIST	Gastrointestinal stromal tumors
JMML	Juvenile myelomonocytic leukemia
LGG	Low-grade gliomas
MEK	Mitogen-activated extracellular signal-regulated kinas
MPNST	Malignant peripheral nerve sheath tumors
MRI	Magnetic resonance imaging
NCR	Netherlands Cancer Registry
NF1	Neurofibromatosis type 1
NIH	National Institutes of Health
NPV	Negative predictive value
OCT	Optical coherence tomography
OPG	Optic pathway gliomas
OS	Overall survival
PALGA	Dutch Pathology Database
PCC	Oheochromocytoma
PGL	Paraganglioma
PN	Plexiform neurofibromas
PPV	Positive predictive value
PRI	Perceptual Reasoning Index
PSI	Processing Speed Index
RCT	Randomized Controlled Trial
SD-OCT	Spectra domain optical coherence tomography
SOBOPE	Brazilian Society of Pediatric Oncology
VA	Visual acuity
VCI	Verbal Comprehension Index
VF	Visual field testing
VMA	Vanillylmandelic acid
WB-MRI	Whole-body magnetic resonance imaging

## Appendix A

**Name**	**Specialty**
Adrialdo José Santos	Clinical neurologist
Adriana Pessoa Mendes Eris	Dermathologist
Alayde Vieira Wanderley	Pediatric Oncology
Ana Karolina Maia	Geneticist
Augusto Elias Mamere	Radiologist
Camila Maia Martin Daiggi	Pediatric Oncology
Carlos Magno Leprevost	Geneticist
Diogo Soares	Geneticist
Dov Charles Goldenberg	Surgeon
Elvis Terci Valera	Pediatric Oncology
Gabriel Batistella	Clinical neurologist
Izabella Costa Santos	Surgeon
Karine Corrêa Fonseca	Pediatric Oncology
Mara Lucia Santos	Pediatric Neurology
Marcia Gonçalves Ribeiro	Geneticist
Marina Cormedi	Geneticist
Mauro Gueller	Immunogeneticist
Nonato Mendonça Lott Monteiro	Pediatric Oncology
Rafael Guerra Cintra	Pediatric Neurology
Rayana Maia	Geneticist
Rebeca Ferreira Marques	Pediatric Oncology
Roberto Bueno	Dermathologist
Victor Evangelhista	Geneticist

## Appendix B

Below are all recommendations that did not reach first round of agreement.

Recommendation 2—It is recommended for all pediatric patients with NF1 to undergo retinal evaluation by optical coherence tomography whenever possible. (75% agreement in the first round).

Recommendation 3—Routine triage with magnetic resonance imaging (MRI) of the central nervous system and optical pathways is not recommended for pediatric patients with asymptomatic NF1. MRI is reserved for patients with recent visual impairment or physical signs such as proptosis, strabismus, nystagmus, persistent headache, precocious puberty, abnormal growth patterns, or other ophthalmologic and/or neurological signs and symptoms suggestive of optic tumors. (50% agreement in the first round).

Recommendation 10—It is recommended for pediatric patients with NF1 and plexiform neurofibromas that are symptomatic or disfiguring, whenever possible, surgical treatment with complete or partial resection of the lesion, which is considered the only curative therapeutic option. (63% agreement in the first round).

Recommendation 19—In cases of unresectable MPNST, partially resected or with large volume (>5 cm), radiotherapy and/or chemotherapy are often incorporated into the treatment plan. (75% agreement in the first round).

Recommendation 20—In case of indication of chemotherapy, it is recommended as first line the combination of ifosfamide and doxorubicin for the treatment of pediatric patients with NF1 and presence of MPNST (56.2% agreement in the first round).
